# GraphPart: homology partitioning for biological sequence analysis

**DOI:** 10.1093/nargab/lqad088

**Published:** 2023-10-16

**Authors:** Felix Teufel, Magnús Halldór Gíslason, José Juan Almagro Armenteros, Alexander Rosenberg Johansen, Ole Winther, Henrik Nielsen

**Affiliations:** Department of Biology, University of Copenhagen, 2200 Copenhagen, Denmark; Digital Science & Innovation, Novo Nordisk A/S, 2760 Måløv, Denmark; Department of Genomic Medicine, Copenhagen University Hospital/Rigshospitalet, 2100 Copenhagen, Denmark; Department of Genetics, Stanford University School of Medicine, Stanford, CA 94305, USA; Department of Biomedical Data Science, Stanford University, Stanford, CA 94305, USA; Department of Computer Science, Stanford University School of Engineering, Stanford, CA 94305, USA; Department of Biology, University of Copenhagen, 2200 Copenhagen, Denmark; Department of Genomic Medicine, Copenhagen University Hospital/Rigshospitalet, 2100 Copenhagen, Denmark; Department of Applied Mathematics and Computer Science, Technical University of Denmark, 2800 Kgs. Lyngby, Denmark; Department of Health Technology, Technical University of Denmark, 2800 Kgs. Lyngby, Denmark

## Abstract

When splitting biological sequence data for the development and testing of predictive models, it is necessary to avoid too-closely related pairs of sequences ending up in different partitions. If this is ignored, performance of prediction methods will tend to be overestimated. Several algorithms have been proposed for homology reduction, where sequences are removed until no too-closely related pairs remain. We present GraphPart, an algorithm for homology partitioning that divides the data such that closely related sequences always end up in the same partition, while keeping as many sequences as possible in the dataset. Evaluation of GraphPart on Protein, DNA and RNA datasets shows that it is capable of retaining a larger number of sequences per dataset, while providing homology separation on a par with reduction approaches.

## Introduction

When constructing a statistical or machine learning (ML) method, it is crucial to test its predictive performance on a test set: a dataset that was not involved in calculating the parameters during the training process. This is necessary to measure whether the method can generalize from the examples in the training set and produce useful output for previously unseen data. If a model reproduces its training examples in too much detail, it uses its parameters to fit not only the common pattern in the data, but also the individual noise in each data point. When this happens, the performance on the test set goes down, and the model is said to be overfitted (1). The common approach to monitor potential overfitting is to split the data into separate sets that are used for training and testing. If the model has hyperparameters such as architecture, learning rate and length of the training process, three datasets should be used: a training set used for optimizing the model parameters, a validation set for optimizing the model architecture and training process and a test set for measuring the performance (2,3).

An extension of this approach to obtain more reliable estimates is *k*-fold cross-validation, where the data is split into *k* partitions so that the training procedure can be repeated multiple times with the training, validation and test sets swapped (3). If all combinations of training and validation set are tried for each test set so that *k* × (*k −* 1) training runs are performed, this is termed nested cross- validation (4).

This problem is not unique to computational biology. In fact, all ML applications rely on estimating performance on test data that was not used for training or optimization. Typically, this can be achieved by splitting the data randomly. However, for biological sequences this approach is severely flawed: Some sequences are evolutionarily related (homologous), and random splitting would lead to close homologs of training sequences being present in the validation and test data. If this is not taken into account, predictive performance can be overestimated. This has been recognized early in the history of bioinformatics (5,6).

The commonly accepted way of dealing with this problem is to reduce the dataset until no pairs of too closely related sequences remain before splitting it into folds (*homology reduction*). But this approach throws away the information represented by the variation between the closely related sequences. As an alternative approach, groups of closely related sequences could be allowed in the dataset, as long as they are placed in the same fold. We will term this approach *homology partitioning*. This requires an algorithm for splitting the data so that no too-closely related pair of sequences end up in different folds.

However, it is non-trivial to define when sequences are neighbors or ‘too closely related’. This question has two parts: which measure of similarity should be used, and what is an acceptable cutoff for this measure of similarity? The standard way of comparing two sequences is to do a pairwise alignment, either global (7) or local (8). Then, the percentage of identical nucleotides or amino acids can be calculated, and this percentage is often used as a measure. However, if local alignment is used, it is necessary to take into account the alignment (overlap) length as well. An 80% identity is not significant if it is just four out of five positions, but if it is 40 out of 50 it is highly significant. This issue can be addressed in several ways: by using a combination of percentage identity and overlap length as the criterion (5,9,10); by using the alignment score instead of percentage identity (9,11,12); by dividing the number of identical letters in the overlap by the length of the shorter sequence (instead of by the length of the overlap) or by using global alignment.

Two widely used algorithms for homology reduction were published by Hobohm and coworkers in 1992 (6). Algorithm 1 (‘select until done’) starts with a list of sequences (which may be ranked according to some quality criterion) and works by selecting the first sequence and removing all its neighbors, then selecting the next sequence from the list and so on, until the list is exhausted. Algorithm 2 (‘remove until done’) works by removing the sequence with the largest number of neighbors first, then recalculating the number of neighbors for each sequence and continuing to remove the sequence with the largest number of neighbors until no pair of neighbors remain. Algorithm 2 typically preserves more sequences in the final set, but requires calculating all pairwise similarities, while Algorithm 1 only requires a subset of the distances.

An alternative to Hobohm's two algorithms is the ‘greedy-min’ algorithm, which selects the sequence with the smallest number of neighbors, removes all its neighbors, recalculates the number of neighbors for each sequence and again selects the sequence with the smallest number of neighbors and repeats until all sequences have either been selected or removed (13). It tends to preserve more sequences than Hobohm's Algorithm 2, but in practical tests, we find that the difference is negligible (results not shown).

As mentioned, a drawback to the Hobohm 2 algorithm is the need to calculate all pairwise distances or similarities, which can be computationally very expensive if full Needleman–Wunsch (7) or Smith–Waterman (8) alignment is performed. To deal with this, a number of faster approximative clustering algorithms have been introduced, notably CD-HIT (14,15) and MMseqs (16,17). They both use a ‘pre-filtering’ step, where short words (*k*-mers) are compared between sequences, and only the most high-scoring pairs are selected for pairwise alignment.

CD-HIT counts the number of *k*-mers in common between each pair of sequences and uses these counts to approximate the percentage of identical residues or nucleotides. Then, it applies a simple greedy incremental clustering, where the sequences are sorted by length, and the longest sequence becomes the representative of the first cluster. After that, each new sequence is compared to the existing clusters and added to the first cluster it matches (i.e. where it has a percentage identity above the threshold to the representative sequence); or if no such match is found, it is designated the representative of a new cluster. When clustering at thresholds below 40% identity (for protein sequences), the PSI-CD-HIT mode is used, which uses BLAST (18,19) for sequence comparisons.

MMseqs differs from CD-HIT in that the *k*-mers are not merely counted but compared using a substitution matrix. On the basis of this comparison, the most high-scoring sequence pairs are selected for local alignment. In MMseqs2 (17), not unlike BLASTP version 2 (19), this alignment step is only performed if there are two *k*-mer matches on the same diagonal. In the clustering step, MMseqs by default uses the greedy set-cover algorithm, where in each step the sequence with the most remaining neighbors is chosen, but it can optionally use the same clustering mode as CD-HIT.

A common feature of these algorithms is that they designate a representative sequence of each cluster and assign a cluster identifier to each sequence. It is important to stress that these algorithms can be used for homology reduction by selecting only the representative sequences, but should not be used for homology partitioning, since the maximum similarity criterion is only applied to the representative sequences, not to the other members of the clusters.

Homology partitioning algorithms are largely missing from the literature. One exception is the recent article by Petti and Eddy about constructing benchmark sets using graph algorithms (20). However, that work focuses on benchmarking remote homology detection methods, so the objective there is to split each pre-aligned family of proteins (as defined by Pfam (21)) into training and test sets, in which no sequence in the test has more than a certain percentage identity to any sequence in the training set. For the best-performing of their proposed algorithms, this was possible for slightly more than half of the full Pfam families. By contrast, our objective is to split an entire dataset, containing both related and unrelated sequences, into subsets (partitions) where no sequence has more than a certain percentage identity to any sequence in any other partition, so that the partitions can subsequently be used in a *k*-fold cross-validation or in a train-validation-test split.

In supervised machine learning tasks, the goal is often to predict a certain functional or structural class (e.g. enzyme class or subcellular location) for each sequence and, thus, each sequence can have a class label. Therefore, a desired additional objective for homology partitioning is to ensure that the relative distribution of labels is approximately the same across all partitions.

In summary, we define the homology partitioning problem as follows: given a set of sequences, a maximum similarity threshold and the number of partitions to generate, find an assignment of sequences to partitions so that (i) no sequence in a partition has identity above the threshold to any sequence in any other partition, and (ii) as many sequences as possible are retained in the partitioned dataset overall. Optionally, if the sequences have class labels, the method should ensure that all partitions have approximately the same number of sequences for each label.

**Figure 1.**
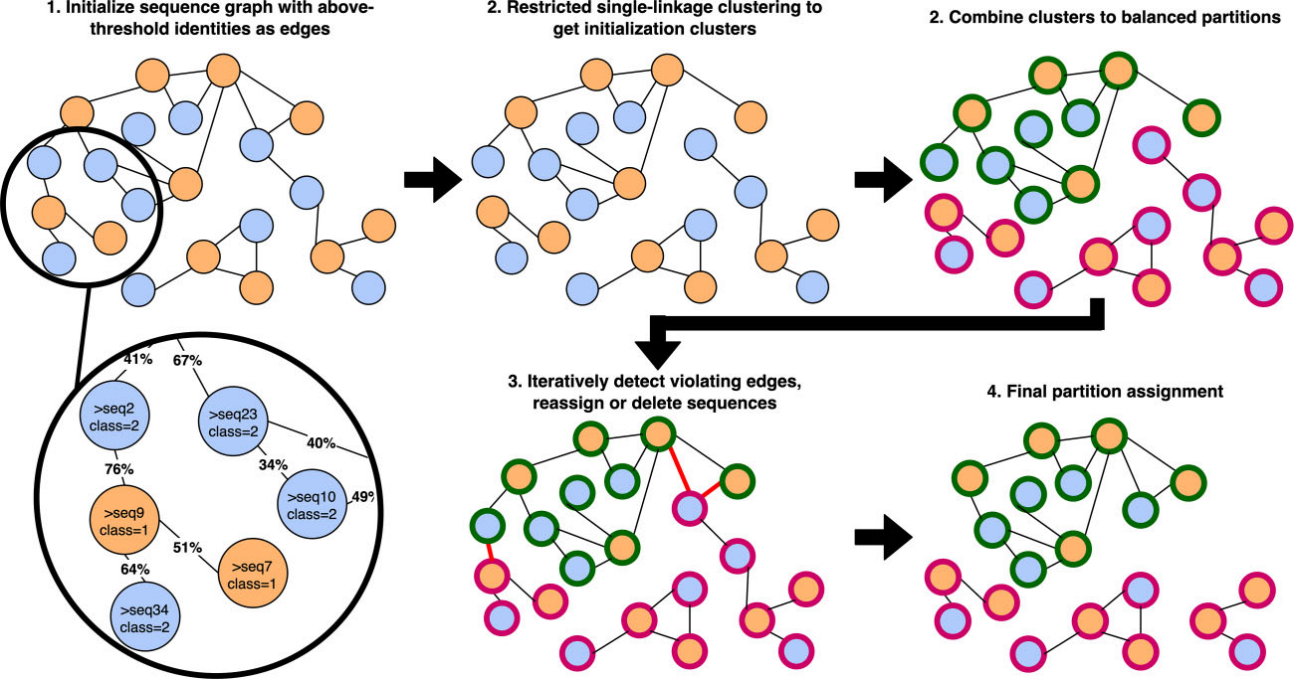
The GraphPart algorithm. Given a graph of sequences of two classes (orange and blue), with edges between sequences indicating identity above a chosen threshold, restricted single-linkage clustering is applied to yield class-balanced clusters. These clusters are combined to yield *k* (=2) balanced even-sized partitions (green and red). Given the partitions and the original sequence graph, sequences are iteratively removed or moved between partitions until there are no edges connecting the partitions, eliminating homology between different partitions.

To address this problem, we developed GraphPart, a graph clustering algorithm that finds the partitions by first finding and merging clusters, followed by an iterative reassignment and removal step (Figure 1). After an initial all-vs-all alignment, GraphPart finds class-balanced clusters of homologous sequences and groups them into the desired number of partitions. Sequences are then iteratively removed, or moved between partitions, until there is no pair of sequences having sequence similarity above the threshold in any two different partitions.

As the similarity measure, we have chosen to use global alignment percentage identity. As mentioned, the measure of choice is often percent identity from a local alignment, where the denominator is not the alignment (overlap) length, but the length of the shorter of the two sequences (e.g. in CD-HIT). A global identity measure will be smaller than or equal to this measure, because the global alignment length is at least as long as the longer of the two sequences.

## Materials and methods

### Datasets

To evaluate the performance of GraphPart in representative applications, we gathered multiple datasets of DNA, RNA and protein sequences (Table 1). All datasets were used in previously published machine learning predictors. For datasets that are already homology reduced in their published versions, we recreate the original datasets according to the instructions laid out in the respective paper. For DNA, we choose datasets of human TATA promoter sequences used for the training of DeePromoter (22) and of DNA regions binding to histone H3 (23,24). For RNA, we recreate the non-coding RNA classification dataset used in nRC (25) by extracting sequences from Rfam (31). As a second dataset, we recreate a dataset used for RNA backbone angle prediction in SPOT-RNA-1D (26) by querying the Protein Data Bank (32).

**Table 1.** Summary statistics of the datasets used for benchmarking

Dataset	Type	Size	Maximum length	Classes	Threshold (%)
DeePromoter (22)	DNA	2929	300	1	80
Histone (23,24)	DNA	14 965	500	2	80
nRC (25)	RNA	6500	758	13	80
SPOT-RNA-1D (26)	RNA	1227	2880	1	80
NetGPI (27)	Protein (truncated)	3618	100	2	30
DeepLoc (28)	Protein	14 004	13 100	11	30
SignalP 5.0 (29)	Protein (truncated)	20 758	70	4	30
NetSurfP 2.0 (30)	Protein	30 046	2128	1	25

For proteins, we investigate performance separately for truncated protein sequences and full proteins. Truncation is often applied when it is already known a priori in which region of the protein the feature of interest is present. We use signal peptides (70 N-terminal amino acids) from SignalP v.5.0 (29) and GPI anchors (100 C-terminal amino acids) from NetGPI (27). For full proteins, we use the eukaryotic subcellular localization dataset from DeepLoc (28) and the secondary structure datasets from NetSurfP v.2.0 (30), which we recreate in its non-reduced state by obtaining Protein Data Bank data at 95% identity from PISCES (33). We partition protein sequences at 30% and RNA sequences at 80% identity. For NetSurfP, as it is a structural prediction task, we use a reduced threshold of 25%. For DNA, there is no commonly applied percentage identity threshold to reduce homology. As 80% is the minimum achievable threshold of CD-HIT-EST, we also use 80% for DNA.

### Baselines

For each dataset, we compare the performance of GraphPart to the commonly used programs CD-HIT (v.4.8.1) (14) and MMseqs2 (v.13.4511) (17). We benchmark two different application modes: homology reduction and homology partitioning. We cluster all sequences to the given identity threshold according to the programs’ tutorials. For protein data with CD-HIT, we first cluster with cd-hit at 90% identity, then at 60% identity, followed by psi-cd-hit to the final threshold. For nucleotides, we cluster with cd-hit-est directly at the final threshold. In all cases, -g is set to 1 for slower, more accurate clustering. For MMseqs2, we run mmseqs easy-cluster with its default parameters to cluster directly at the given identity threshold. MMseqs2 supports different clustering modes. In our initial experiments, we found no clear effect of the clustering mode on separation quality, so we use the default mode 0 (greedy set-cover) in all experiments.

In the homology reduction mode, we only retrieve the representative sequence for each cluster and partition this reduced set of sequences using the StratifiedKFold method included in Scikit-learn (34). For homology partitioning, we assign all sequences in a cluster to a partition using a heuristic assignment algorithm. We iterate over all clusters, assigning each cluster to a partition so that class label ratios and partition sizes are as balanced as possible. We note that the latter approach is not expected to result in strict homology separation, but is still capable of reducing similarity between partitions compared to random split strategies.

For homology reduction, we additionally benchmark the Hobohm 2 algorithm. We use Needleman–Wunsch alignments computed by needleall from the EMBOSS (35) package (v.6.6.0.0) with the respective defaults for protein and nucleotide sequences, or full all-vs-all alignments without prefiltering or e-value filtering from MMseqs2.

### GraphPart algorithm

To identify homology partitions in a dataset of labeled sequences at a required sequence identity threshold, GraphPart operates in a four-step procedure. We give a brief overview of the steps next and further discuss each step in detail.

- **Alignment**. A graph of all sequences is constructed by computing all pairwise alignments using a suitable alignment program, with the resulting percent sequence identities (homology) becoming the graph's edge weights.
- **Clustering**. Clusters of homologous sequences are obtained by applying a restricted version of agglomerative single-linkage clustering that
  - uses a predefined similarity threshold,
  - will not merge clusters if the resulting cluster becomes larger than the expected size of a partition and
  - will not merge clusters if the resulting count of any class becomes larger than its expected count per partition.
- **Partitioning**. The clusters are iteratively assigned to the partitions such that the number of sequences of any label is approximately the same across partitions.
- **Separation**. Finally, a heuristic is applied to iteratively reassign or remove sequences until no sequences belonging to different partitions have an identity violating the threshold.

#### (i) Alignment

GraphPart needs to compute all pairwise sequence identities in the input dataset *S*. For exact Needleman-Wunsch alignments, we use needleall from the EMBOSS (35) package. When using MMseqs2 (17) alignments, we compute all-vs-all alignments without a prefiltering step as documented in the MMseqs2 user guide. We return all identities that exceed the given identity threshold, without filtering for e-values or for alignment coverage. As the default denominator for sequence identity (length of the local alignment including gaps) can give misleading results when not using coverage controls, we use the length of the shorter sequence as the denominator. When aligning nucleotide sequences, the prefiltering step cannot be omitted and is thus executed with the sensitivity set to maximum.

The GraphPart partitioning algorithm operates on real-valued distance metrics. Sequence identities ranging from 0 to 1 are converted to distances as d(*a*,*b*) = 1-identity(*a*,*b*). The partitioning threshold undergoes the same conversion. Alternatively, GraphPart can accept any precomputed distance or similarity metric and skip the alignment step.

The result of this step is a weighted graph, with a set of nodes, *S*, and set of weighted edges, *D*. Each node *s* ∈ *S*, represents an input sequence and each weighted edge *d* ∈ *D* is the distance between the two nodes connected by the edge. Each input sequence has one label *l* ∈ *L*, where *L* is the set of all possible labels and *S_l_* is the set of nodes labeled with label *l*.

#### (ii) Clustering

The sequences are then clustered using a restricted form of single linkage clustering. Each *s* ∈ *S* is initially regarded as a separate, or singleton, cluster. *D* is sorted in ascending order and iterated over to connect pairs of clusters, forming larger clusters. If a connection between two clusters would result in a cluster whose size exceeds the expected partition size $\frac{{| S |}}{k}$, where *k* is the number of partitions, the clusters are not combined. Likewise, if the connection would result in a cluster having more sequences of any class label *l* ∈ *L*, than the expected value $\frac{{| {{S}_l} |}}{k}$, the clusters are not combined. If a pairwise distance exceeds the threshold, the procedure is stopped. The result of this step does not connect all homologous sequences. Rather, it serves as an initial grouping of sequences to assemble balanced partitions, which are refined to achieve the final threshold.

#### (iii) Partitioning

The clusters generated in the previous step are partitioned for balanced class label composition. The desired number of partitions are initialized as empty sets. The list of clusters, in the order in which it is generated by step (ii), is iterated over one by one, and all sequences of a cluster are assigned to one of the partitions. The partition to be assigned to is chosen so that the imbalance in the number of sequences of a class label *l* across partitions is minimized after assignment. The result of this step is a partitioning of all sequences into *k* class-balanced partitions. Owing to the restriction of step (ii), the partitions can have threshold-violating distances between each other.

#### (iv) Separation

The last step is the removal phase, for which pseudocode is given in Supplementary Code 1. Sequences that have a distance lower than the threshold to any sequence in another partition are iteratively removed from *S* together with the corresponding pairwise distances being removed from *D*.

To achieve this, the following procedure is repeated starting from iteration *i* = 1: For each sequence *s* ∈ *S*, the number of connections below the threshold to its own partition and to the other partitions is computed. If *s* has more connections to sequences in another partition than in its own, *s* is moved to that partition. Optionally, this moving step can be skipped. While iterating over *S*, we compute *c*, the total number of sequences having cross-partition connections below the threshold. After processing all *s* ∈ *S*, the $c\times\log_{10}( {i/100} ) + 1$ sequences with most cross-partition connections below the threshold are removed from *S*. The multiplier with which we multiply *c* is a heuristic scaling factor that ensures that removal proceeds less aggressively in early iterations. In early iterations, where interconnectivity is high, as most connecting sequences still remain in the graph, removal is done more conservatively, as removing few high-connectivity sequences initially can change the cross-partition connectivity of many other sequences. Removing many sequences simultaneously with high-connectivity sequences is therefore avoided to potentially preserve them in later iterations. With increasing iterations, we let the removal step proceed more aggressively, as removal on a larger scale seems necessary for finding a valid solution.

The procedure is repeated as long as $c >0$, incrementing *i* with $ + 1$ after each iteration. The result of this step is a partitioning of all sequences into *k* partitions that have no threshold-violating distances between each other. As we strictly require *c* to be 0, removal can result in the class balance of partitions deteriorating compared to the balance achieved in step (iii), or, in extreme cases, even the removal of complete partitions. In GraphPart, achieving separation takes precedence over class balancing.

GraphPart also accepts binary priority labels to indicate which sequences should be removed first, if removal is required. This can be used when, for example, a subset of sequences is of higher quality and should preferentially be retained in the final dataset. When using such labels, the removal procedure is first performed in a restrained mode, only removing low-priority sequences until there are no more cross-partition connections for this group. If there are still cross-partition connections remaining, the removal procedure is then repeated in the unrestrained mode.

In addition to finding balanced *k*-fold partition assignments, GraphPart is capable of generating train-validation-test splits, e.g. reserving 10% of the data for testing and 5% for model validation. To achieve this, we first partition the data into *n* partitions, with *n* being a number such that it yields natural numbers when multiplied with both the test and the validation ratios. We select the maximally connected *k* of the *n* partitions to yield the train set, with $k\ = \ n\times ( {100\% - \ test \% \ - validation \% } )$. Maximum connectivity is computed by counting all cross-partition edges exceeding the threshold. The same is then done with the remaining unassigned partitions to yield the test set. The final remainder becomes the validation set. After reassignment of all sequences according to their new split memberships, we proceed with the removal phase as in the standard case. Depending on the dataset, this procedure can result in the retention of more sequences, compared to e.g. performing a 10-fold split and then combining partitions to yield the desired ratios (Supplementary Figure S1).

### Evaluation

For evaluating homology separation quality, we compute the maximum cross-partition pairwise identity for each sequence. Successful separation implies that there is no sequence in the dataset that has a cross-partition pairwise identity higher than the defined threshold.

We use EMBOSS needleall (35) for pairwise Needleman–Wunsch global alignments. Given a partition assignment, we align each sequence in a partition to all sequences in any other partition to find the sequence's maximum cross-partition pairwise sequence identity. We leave all alignment parameters at their defaults, using EBLOSUM62 for protein and EDNAFULL for nucleotide alignments.

To evaluate runtime performance, GraphPart was executed on a 12-core Intel Xeon Gold 6126 HPC node, with the full node reserved for GraphPart execution for comparability. Execution times were logged directly by the GraphPart Python script. For both the sequence length and dataset size runtime evaluations, we upsampled the NetGPI dataset. For size, we sampled sequences with replacement to the desired total number. For length, we first trimmed the sequences to 50 amino acids and repeated the trimmed sequence *N* times to yield sequences of the desired length. For this experiment, we ran graphpart needle in –triangular mode, meaning that only the upper triangle of the similarity matrix is computed. The runtimes of GraphPart reported for the individual benchmark datasets were measured on a 64-core node equipped with Intel Xeon E5-4660 v.4 CPUs.

For benchmarking sequence retention, we compare GraphPart to the Hobohm 2 algorithm. At any given threshold, we count the number of sequences remaining in the dataset. GraphPart was run for five partitions.

## Results and discussion

**Figure 2.**
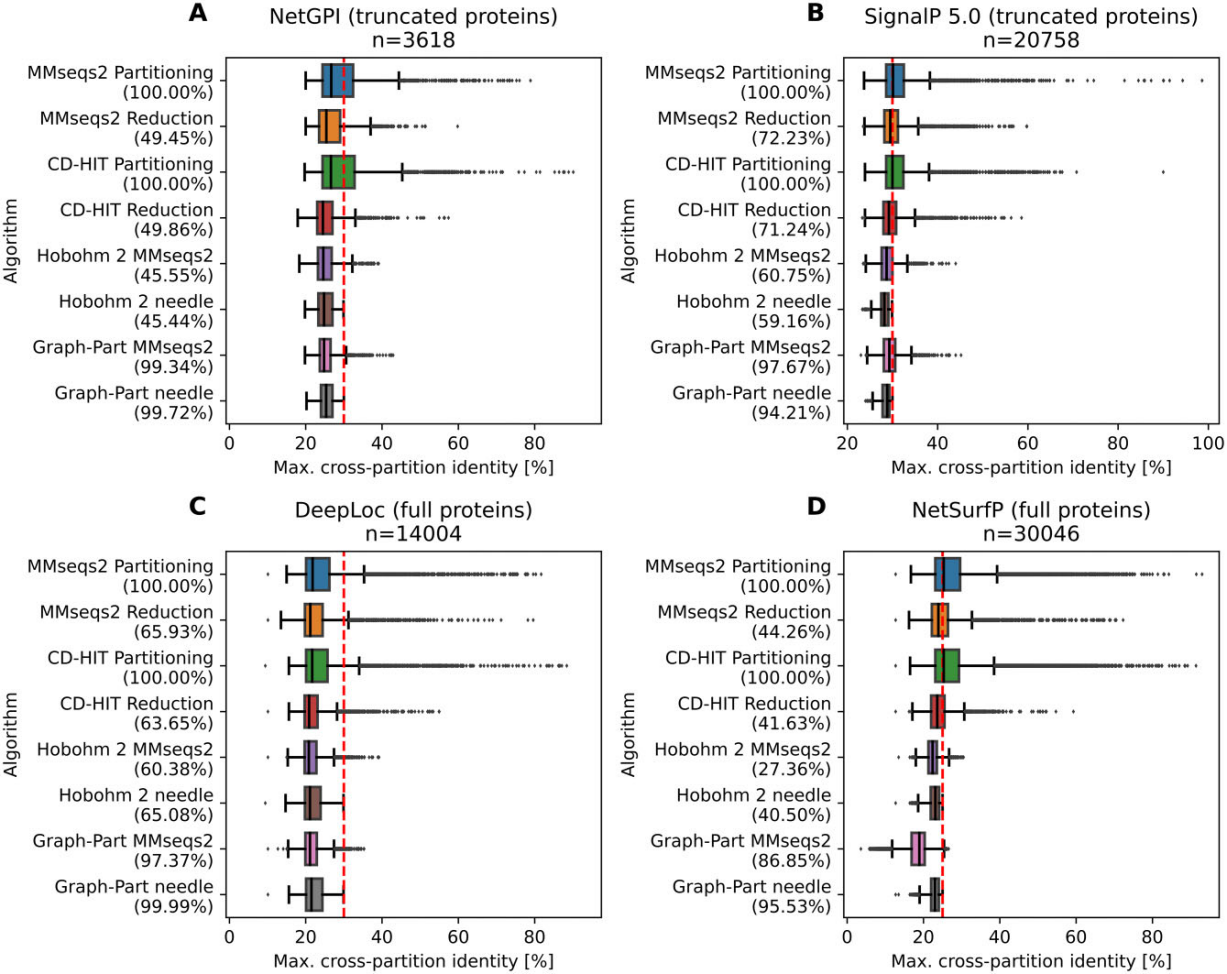
Benchmarking results on protein datasets. Datasets were partitioned into five folds. The target pairwise identity threshold is indicated as the red dashed line. All algorithms were run for the generation of five equal-sized balanced partitions. Partitioning refers to first running clustering at the given threshold, followed by assignment of all sequences of a cluster to one fold. Reduction refers to only taking the representative sequence of each cluster, and grouping those into folds. The GraphPart algorithm and Hobohm 2 were executed using the two implemented alignment algorithms. The numbers in parentheses are the fractions of sequences retained in the partition assignment.

Throughout the investigated datasets, we find that performing homology partitioning with assignment of homology clusters into partitions fails to achieve homology separation (Figure 2). This result is expected, as the procedure essentially only maximizes similarity within clusters in a partition, rather than minimizing similarity between partitions. However, we also find that homology reduction using the baseline strategies, which is expected to minimize similarity, can leave a considerable amount of cross-partition NW identities above the threshold.

In all investigated datasets, reduction with default parameters by CD-HIT yields better results than MMseqs2, but still falls short of achieving complete separation. Moreover, we observe a dependency on sequence length: Both MMseqs2 and CD-HIT see a substantial decrease in separation quality when using shorter, truncated sequences instead of full proteins (Figure 2A, B). As expected, GraphPart and Hobohm 2 achieve perfect separation irrespective of sequence length when using NW alignments. Additionally, using GraphPart or Hobohm 2 with MMseqs2 alignments results in better separation than directly applying MMseqs2 or CD-HIT for homology reduction.

**Figure 3.**
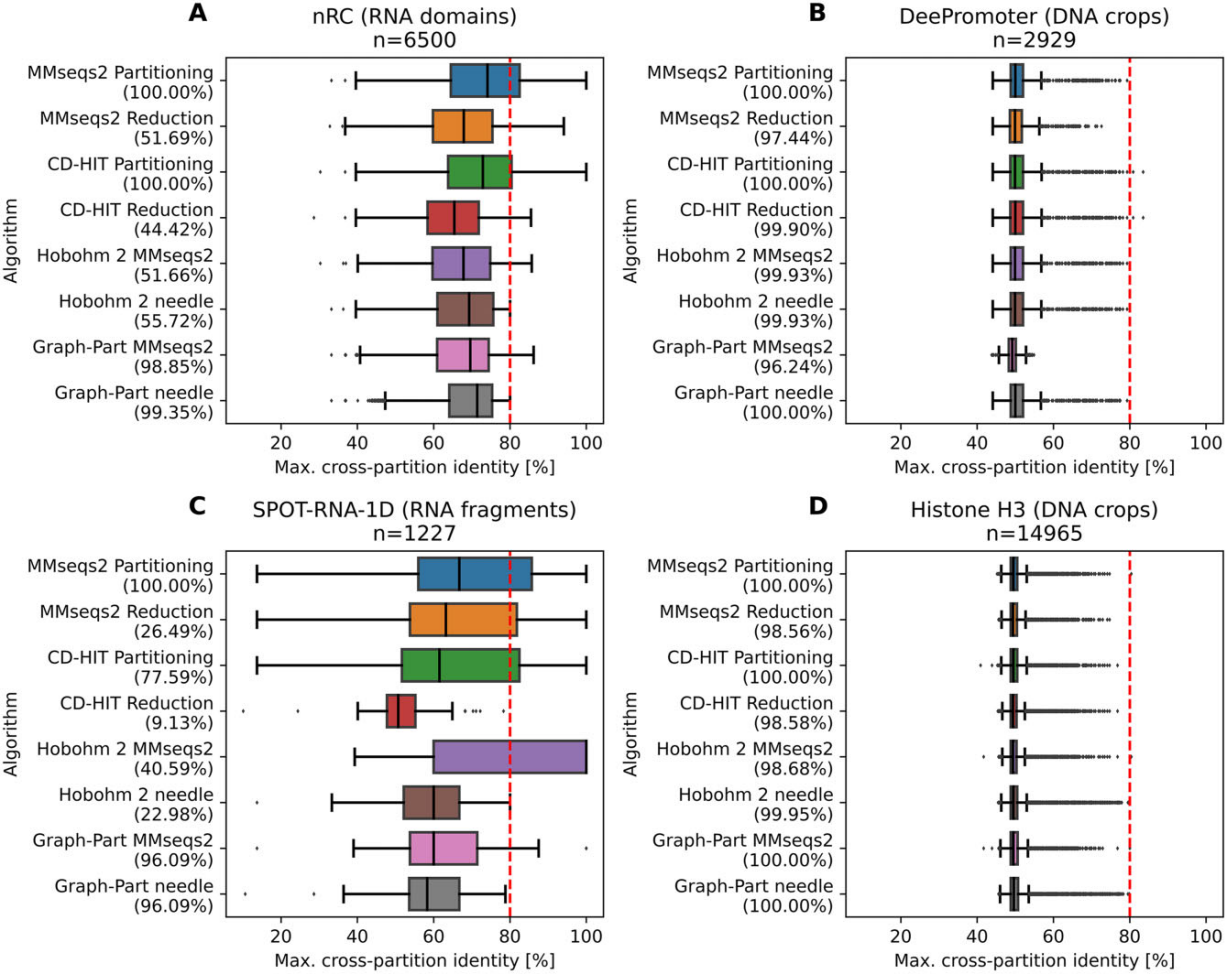
Benchmarking results on nucleotide datasets. Datasets were partitioned into five folds. The target pairwise identity threshold is indicated as the red dashed line. All algorithms were run for the generation of five equal-sized balanced partitions. Partitioning refers to first running clustering at the given threshold, followed by assignment of all sequences of a cluster to one fold. Reduction refers to only taking the representative sequence of each cluster, and grouping those into folds. The GraphPart algorithm and Hobohm 2 were executed using the two implemented alignment algorithms. The numbers in parentheses are the fractions of sequences retained in the partition assignment.

When benchmarking on nucleotide data, we observe that MMseqs2 homology reduction can fail to remove identities above the threshold (Figure 3). Overall, RNA datasets show a higher redundancy than our protein datasets, with homology reduction removing the majority of the data. Still, GraphPart retains more than 95% of all sequences while achieving perfect separation. Furthermore, for DNA datasets we find that a threshold of 80% maximum identity, the minimum that is achievable with CD-HIT-EST, is trivially achievable by all baseline algorithms without loss of sequences. On these datasets, random partitioning of sequences without taking sequence identities into account achieves good homology separation already (Supplementary Figure S2).

**Figure 4.**
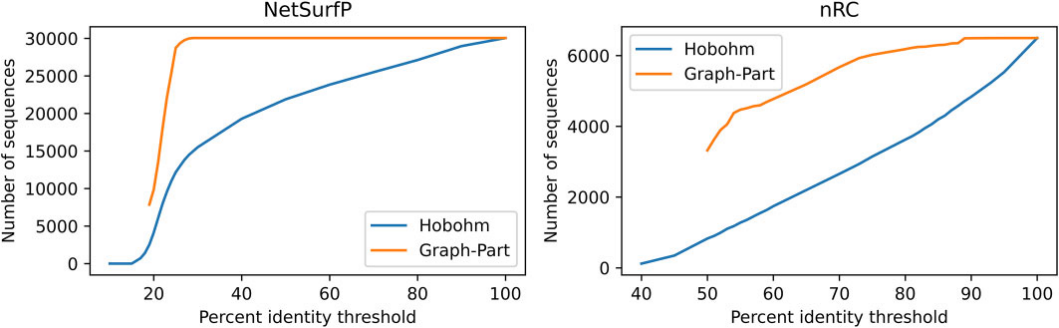
The number of sequences retained at a given maximum percentage pairwise identity threshold for GraphPart and the Hobohm 2 algorithm. Needleman–Wunsch alignments were used for both algorithms, GraphPart was run for five partitions. At very low identities, GraphPart fails as heuristic iterative removal leads to the deletion of complete partitions.

Besides achieving separation on par with Hobohm 2 homology reduction, GraphPart is capable of retaining more than 50% more sequences in its partitions on some datasets. This is due to GraphPart only removing sequences until there are no more pairwise identities between different partitions, rather than removing sequences until the pairwise threshold is obeyed by all remaining sequences in homology reduction. We further investigate the retention capability by benchmarking against Hobohm 2 with NW alignments at multiple thresholds (6). While Hobohm 2 continuously reduces the number of sequences with decreasing identity thresholds, GraphPart retains most sequences until a critical threshold is reached at which large-scale removal becomes unavoidable and eventually fails to partition the dataset into *k* folds as complete folds are removed (Figure 4). In practice, we find that this critical threshold lies lower than commonly used homology cutoffs of 20 to 30% identity for protein sequences or 80% identity for nucleotide sequences.

GraphPart arrives at a reasonably class-balanced partitioning for most datasets (Supplementary Figure S3). In some cases, the moving step of the algorithm can cause one partition to grow larger at the expense of others. If this is not wanted, it could be alleviated by removing sequences from the partitions deemed too large, either at random or using e.g. the Hobohm 2 algorithm. On the nRC dataset, we observe that GraphPart fails to distribute the ribozyme class evenly between partitions.

The size of each individual partition will be roughly inversely proportional to the number of partitions (*k*), but not exactly. One reason is, as mentioned before, the moving step, another is that a higher *k* may necessitate removal of more sequences to achieve perfect separation between all partitions. We have investigated this empirically for two of the datasets by varying *k* from 2 to 20 (Supplementary Figure S4) and found that although the number of removed sequences does indeed increase with *k*, the effect is not drastic.

## Limitations

While GraphPart inherently suffers from quadratic complexity due to computing a full all-vs-all similarity matrix in step 1 (Supplementary Figure S5), many supervised machine learning datasets fall within a range of size and sequence length similar to the benchmarked datasets, in which application of Needleman–Wunsch alignments is feasible when using multiple cores in parallel (Supplementary Table S1), or using Graphpart with fast MMseqs2 alignments. Large-scale applications with orders of magnitude more sequences—such as protein language models—often have a different objective than supervised learning, e.g. representation learning, and are beyond the scale amenable to homology separation methods relying on all-vs-all alignments.

We find that GraphPart succeeds at partitioning a range of datasets at previously used identity thresholds, but can fail when further reducing thresholds to very low values as the partitioning problem becomes too difficult for GraphPart's heuristic algorithm (Figure 4). In general, the difficulty of a partitioning problem is highly dependent on the chosen threshold and the number of partitions as well as on the intrinsic properties of a dataset, such as sequence redundancy, the label imbalance and the number of labels. On datasets where these properties are high, GraphPart can fail to find a partitioning solution. Homology reduction algorithms then have to be used instead, leaving room for future improvement of homology partitioning algorithms.

## Conclusion

We introduce GraphPart, a dedicated sequence homology partitioning algorithm addressing the need of homology separation into *k* sets in machine learning on biological sequence data. GraphPart shows superior performance over reduction-based separation approaches by preserving more sequences in its partitions. Additionally, it allows for balancing of partitions for additional criteria while achieving homology separation. By utilizing exhaustive global Needleman–Wunsch alignments, GraphPart guarantees separation at its chosen identity threshold. Additionally, other alignment programs such as MMseqs2 can be used by GraphPart and show competitive performance while greatly improving speed.

## Supplementary Material

lqad088_Supplemental_File

## Data Availability

GraphPart can be installed as a Python package from https://pypi.org/project/graph-part/. An online version is available at https://ku.biolib.com/graphpart. The source code is available on Zenodo at https://doi.org/10.5281/zenodo.8354390 and on GitHub at https://github.com/graph-part/graph-part. All datasets and the baseline scripts used for benchmarking are included in the repository.
