# Supplementary material for: GraphPart: homology partitioning for biological sequence analysis
Source: NAR Genom Bioinform. 2023 Oct 16;5(4):lqad088. doi: 10.1093/nargab/lqad088 (PMC10578201; doi:10.1093/nargab/lqad088)
Supplement: lqad088_Supplemental_File [file lqad088_supplemental_file.pdf]

Supplementary Information for GraphPart: Homology partitioning for biological sequence analysis

Felix Teufel[1,2], Magnús Halldór Gíslason[3], José Juan Almagro Armenteros[4,5], Alexander Rosenberg Johansen[6], Ole Winther[1,3,7], and Henrik Nielsen[8,*]

[1]Department of Biology, University of Copenhagen, Copenhagen, Denmark
[2]Digital Science & Innovation, Novo Nordisk A/S, Måløv, Denmark
[3]Department of Genomic Medicine, Copenhagen University Hospital/Rigshospitalet, Copenhagen, Denmark
[4]Department of Genetics, Stanford University School of Medicine, Stanford, CA 94305, USA
[5]Department of Biomedical Data Science, Stanford University, Stanford, CA 94305, USA
[6]Department of Computer Science, Stanford University School of Engineering, Stanford, CA 94305, USA
[7]Department of Applied Mathematics and Computer Science, Technical University of Denmark, Kgs. Lyngby, Denmark
[8]Department of Health Technology, Technical University of Denmark, Kgs. Lyngby, Denmark
*To whom correspondence should be addressed, email: henni@dtu.dk

Contents:

- Code 1
- Supplementary Figures 1-5
- Supplementary Table 1

```
Inputs: S, D, threshold
i = 1
while c>0:
	connected = []
	for s in S:
		c_other = count_cross_partition_connections(s, S, D, threshold)
		c_own = count_own_partition_connections(s, S, D, threshold)
		if c_other > c_own:
			S = move_to_closer_partition(s,S)
		c_other = count_cross_partition_connections(s, S, D, threshold)
		if c_other > 0:
			connected.append(s)

	c = length(connected)
	n_to_remove = c * log10(i)/100 + 1
	S, D = remove_entities(S, D, connected, n_to_remove)
	i = i + 1
```

Code 1. Pseudocode of the iterative removal procedure. **S** is a structure containing all entity identifiers and their associated partition membership labels. **D** contains the distances between all elements of `S`. `threshold` is a float value. The `move_to_closer_partition` operation can be disabled.

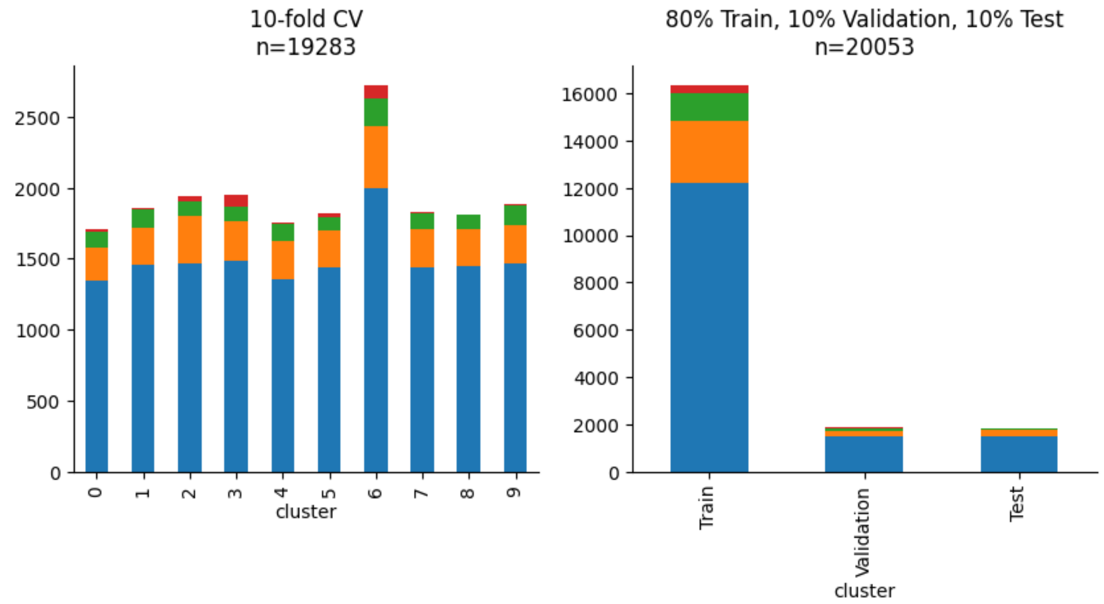

Supplementary Figure 1. Train-Validation-Test split mode of GraphPart. The SignalP dataset was once partitioned with *k*=10, and once with a validation ratio and a test ratio of 10%. In the *k*=10 split, the percent identity threshold is enforced between all 10 partitions, requiring the removal of sequences. The Train-Validation-Test split mode first also creates 10 partitions, but then groups them into the 3 required partitions before enforcing the constraint. As separation of 3 partitions is easier than separation of 10 partitions, less sequences are removed. We

executed the mode with `--no-moving`, as the moving step reduces the number of minority class sequences in the Test partition too much. The 10-fold split was executed regularly, including the moving operation.

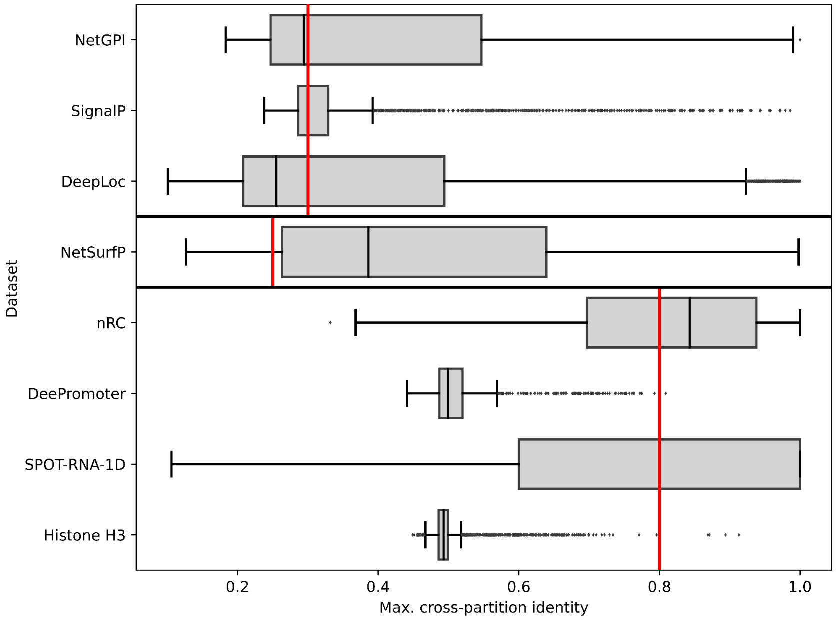

Supplementary Figure 2. Performance of a random baseline for homology partitioning. Sequences were partitioned into five folds using `StratifiedKFold` from Scikit-learn without using any sequence identity information. The red lines indicate the target pairwise identity threshold for the respective datasets.

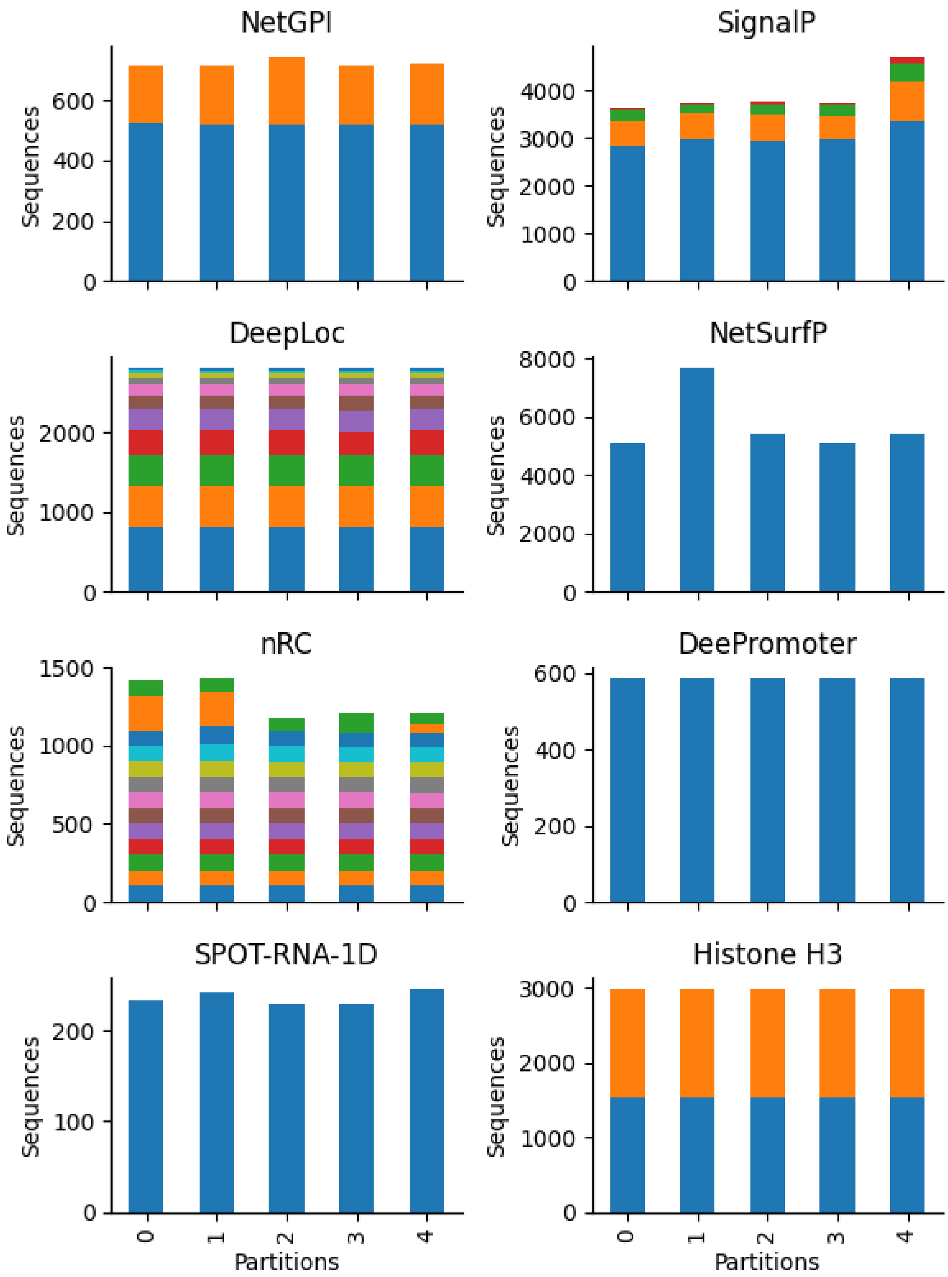

Supplementary Figure 3. Class label distributions in the GraphPart needle partitions. This figure is based on the same runs that are shown in Figures 2 and 3. In the nRC dataset, we find that class 10 (*ribozymes*) has high connectivity, so GraphPart fails to distribute it evenly and the majority of sequences get assigned to two partitions. There is only one ribozyme sequence remaining in partitions 2 and 3 each.

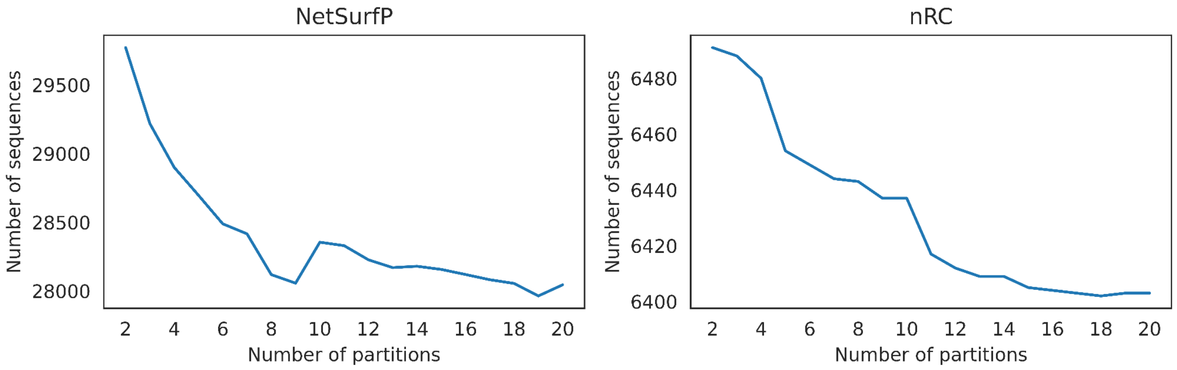

Supplementary Figure 4. The number of sequences retained at a given number of partitions *k*. Needleman-Wunsch alignments were used. With increasing *k*, the partitioning problem becomes more challenging, requiring the removal of more sequences to achieve separation.

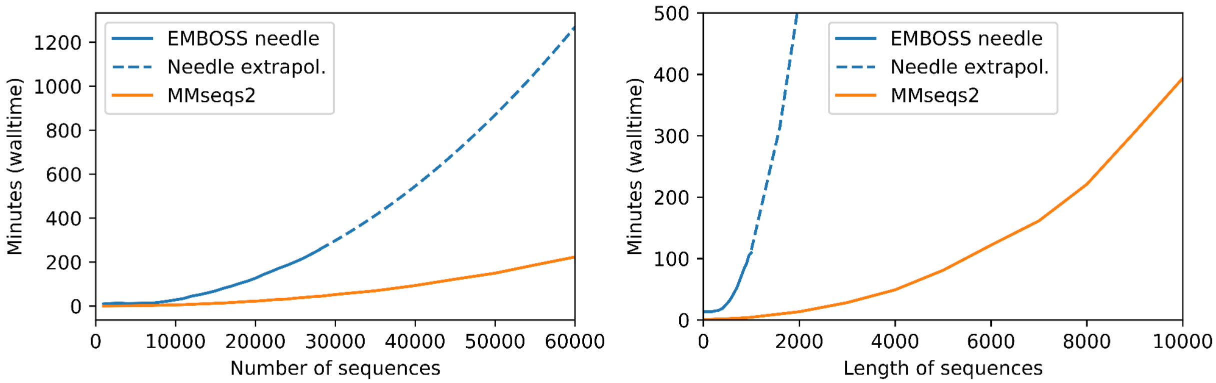

Supplementary Figure 5. Time complexity of GraphPart. Due to the exhaustive computation of pairwise alignments, GraphPart scales quadratically with the number of input sequences. With regards to sequence lengths m and n, pairwise alignment algorithms have a time complexity of O(mn). For the investigated dataset sizes, the execution time of the actual partitioning algorithm itself is negligible compared to the time spent on pairwise alignments. Data was prepared from the NetGPI dataset as described in the methods, using sequences of maximum length 100 for the input size benchmark, and using 3,618 inputs for the length benchmark.

[minutes: seconds]	GraphPart needle		GraphPart mmseqs2		CD-HIT partitioning	CD-HIT reduction	MMseqs2 partitioning	MMseqs2 reduction	Hobohm 2 needle	Hobohm 2 MMseqs2
Dataset	Alignment	Partitioning	Alignment	Partitioning					Partitioning	Partitioning
DeePromoter	10:15	0:01	0:02	0:01	0:10	0:10	0:32	0:30	0:00	0:00
Histone	431:10	0:01	0:11	0:01	1:03	1:01	0:37	0:21	0:00	0:00
nRC	37:38	0:12	0:04	0:16	0:03	0:02	0:35	0:21	0:00	0:00
SPOT-RNA-1D	0:56	0:00	0:02	0:00	0:01	0:00	0:27	0:21	0:00	0:00
NetGPI	9:57	0:02	0:04	0:04	0:30	0:28	0:10	0:09	0:00	0:00
DeepLoc	499:11	0:01	3:57	0:31	14:34	13:02	0:19	0:15	0:02	0:02
SignalP 5.0	291:22	0:48	0:44	0:37	3:19	3:23	0:16	0:11	0:06	0:05
NetSurfP 2.0	1085:36	12:00	7:47	15:17	9:24	9:12	0:19	0:13	0:17	0:30

Supplementary Table 1. GraphPart runtime. Each dataset was split at k=5, balancing for labels. The alignment runtimes for Hobohm 2 are identical to the GraphPart alignment runtimes, as the same edge lists need to be computed. In the MMseqs2 partitioning/reduction runs, MMseqs2 is executed as `mmseqs easy-cluster`. On some small datasets, this iterative procedure is slower than the MMseqs2 all-vs-all alignment used in GraphPart.
